# Interactive Effects of Copper and Functional Substances in Wine on Alcoholic Hepatic Injury in Mice

**DOI:** 10.3390/foods11162383

**Published:** 2022-08-09

**Authors:** Xiangyu Sun, Jiaqi Wang, Qian Ge, Caihong Li, Tingting Ma, Yulin Fang, Jicheng Zhan

**Affiliations:** 1 Beijing Key Laboratory of Viticulture and Enology, College of Food Science and Nutritional Engineering, China Agricultural University, Beijing 100083, China; 2 Shaanxi Provincial Key Laboratory of Viti-Viniculture, Viti-Viniculture Engineering Technology Center of State Forestry and Grassland Administration, Shaanxi Engineering Research Center for Viti-Viniculture, Heyang Viti-Viniculture Station, Ningxia Eastern Foot of Helan Mountain Wine Station, College of Enology, College of Food Science and Engineering, Northwest A&F University, Xianyang 712100, China; 3 Quality Standards and Testing Institute of Agricultural Technology, Ningxia Academy of Agricultural Sciences, Yinchuan 750002, China

**Keywords:** copper, wine, polyphenols, liver, liver protection, alcoholic damage

## Abstract

This study analyzed the interaction between copper and functional substances in wine under different drinking amounts on alcoholic liver injury in mice. When the daily drinking amount reached 500 mL/60 kg/day (14% abv) with just ethyl alcohol, the liver aspartate aminotransferase, alanine aminotransferase, and total triglyceride levels of mice were significantly increased to 130.71 U/L, 37.45 U/L, 2.55 U/L, the total antioxidant capacity, catalase, and glutathione level decreased significantly to 1.01 U/mL, 30.20 U/mgprot, and 2.10 U/mgprot, and the liver became gradually damaged. Wine could alleviate and reduce the damage caused by ethyl alcohol well. Low concentrations of copper (0.33, 0.66 mg/L) in wine hardly caused hepatic injury in mice and only significantly improved the aspartate aminotransferase values (109.21 U/L, 127.29 U/L) of serum. Combined with the staining evidence, in the case of medium and high intragastric doses (≥500 mL/60 kg/day), 0.99 mg/L copper (the maximum allowed by China’s national standards) in wine began to damage the liver, indicating that under this concentration, the damage of copper to the liver had begun to exceed the protective effect of wine’s functional substances on alcoholic hepatic injury. At all experimental doses, high concentrations (1.33 mg/L, 2.00 mg/L) of copper significantly aggravated alcoholic hepatic injury in mice, indicating that high concentrations of copper have a great toxicological risk. In the future, it is necessary to further strengthen the control of copper content in wine and the inspection of market wines in order to protect the health of consumers.

## 1. Introduction

Chronic alcohol consumption, which is highly prevalent worldwide, is one of the main factors contributing to the global burden of disease. Ethyl alcohol can cause metabolic disorders in multiple organs [1], and long-term drinking can easily lead to steatosis, steatohepatitis, and eventually cirrhosis and hepatocellular carcinoma [2]. At present, alcoholic liver disease (ALD) has become the second most common liver disease, followed by viral liver disease, in the world, and it has shown an increasing trend year by year [3]. The direct toxic effects of ethyl alcohol, oxidative stress, inflammation, gut permeability, and gut microbiota are all factors belonging to the pathogenesis of ALD [4]. Meanwhile, studies have shown that the drinking amount is also closely related to the onset of ALD [5]. 

Multiple epidemiological and experimental studies have shown that moderate and chronic consumption of wine is beneficial to health [6]. This effect could be attributed to the functional components in wine, mainly polyphenols, such as resveratrol [7] and quercetin [8]. Kasdallah-Grissa et al. (2007) proved that resveratrol could avoid the oxidative damage induced by chronic ethanol administration through inhibiting hepatic lipid peroxidation and ameliorated the superoxide dismutase (SOD), glutathione peroxidase (GPx) and catalase (CAT) activities in the liver [9]. Jiang et al. (2016) found that anthocyanins also have liver-protective effects. However, there is still controversy on whether wine could alleviate alcoholic hepatic injury caused by long-term drinking [10,11].

At the same time, copper is the most concerning heavy metal in the wine industry [12,13]. Copper plays an important and positive role in organisms in a narrow low concentration range, but beyond the beneficial range, it will inhibit and even lead to toxicity in cells [14]. Although relevant regulations have been issued (EC No. 1410/2003, OIV, 2013), it is not uncommon for the copper content in grapes and wine to exceed the standard [15,16]. The available evidence shows that chronic copper exposure reduced the antioxidant and immune function of young people [17], and acute copper exposure caused acute gastritis [18]. Despite reports that good-quality wine did not pose toxicological risks [14], and common consumers would not be exposed to dangerous copper levels [19], there is still a lack of research on whether chronic wine consumption with excessive copper would cause the accumulation of copper or aggravates the ALD.

Hence, this study compared the changes in liver indicators, serum indicators and copper contents of mouse livers under different drinking amounts of alcohol, wine and copper-containing wine for a long time, so as to analyze and compare the protective effect of wine on alcoholic hepatic injury in mice under different intake conditions, to explore whether copper in wine will aggravate alcoholic hepatic injury caused by chronic alcohol intake, and further analyze the interaction between copper pollution in wine and wine polyphenols on alcoholic hepatic injury in mice. This research aimed to better understand the impact of copper pollution on wine production, provide a theoretical basis and support for the further development of wine, and help the wine industry towards better development.

## 2. Materials and Methods

### 2.1. Laboratory Animals and Reagents

The animal experiment was approved by the Experimental Animal Care Ethics Committee of Northwest A&F University. A total of 320 ICR mice (male, 9 weeks old) with an average weight of 33 ± 2 g, SPF grade, were purchased from Vital River Laboratory Animal Technology Co., Ltd. (Beijing, China) (Approval Number: SCXK (Beijing) 2012-0001). Before the experiment, all experimental animals were bred adaptively in the animal room for one week. The conditions in the animal room were controlled as follows: the humidity was set to 55% ± 5% and the temperature was set to 25 ± 2 ℃, a 12-h circadian rhythm was ensured, and the experimental animals could drink water and eat freely. After all the mice had adapted to the environment, the experiment was started.

The aspartate aminotransferase (AST) kit, the alanine aminotransferase (ALT) kit, the total bilirubin (TBIL) kit, the alkaline phosphatase (ALP) kit, the total triglyceride (TC) kit, the total cholesterol (TG) kit, the total antioxidant capacity (T-AOC) kit, the catalase (CAT) kit, the glutathione (GSH) kit, the superoxide dismutase (SOD) kit, and the malonaldehyde (MDA) kit were purchased from Nanjing Jiancheng Bioengineering Institute (China).

### 2.2. Intragastric Fluid

“Academic” red wine (Cabernet Sauvignon) (14.0%vol) was provided by the wine technology development center of China Agricultural University, which contained 0 mg/L initial copper content. We added CuSO_4_·5H_2_O to make the wine reach the set copper concentration (0.00 mg/L, 0.33 mg/L, 0.66 mg/L, 0.99 mg/L, 1.33 mg/L, 2.00 mg/L), and it was stored at 4 °C for later use. The control group used saline. Alcohol was prepared at the same ethyl alcohol intake as wine.

Analysis of the phenolic and organic acid composition of intragastric fluid was conducted using a Waters Alliance HPLC 2695 (Waters, Milford, CT, USA) equipped with a photodiode array detector 2996 (Waters, Milford, CT, USA). Separation was performed using a Capcell Pak C18 column (250 mm × 4.6 mm, 5 μm) (Shiseido, Osaka, Japan). The injection volume was 10 μL, and the peak area external standard method was used. The flow rate was 0.5 mL/min. 

The column temperature of phenolic was 28 °C and the detection wavelength was 280 nm. The mobile phase consisted of solvent A (V(water):V(acetonitrile) = 19:1, containing 0.3% acetic acid), and solvent B (V(acetonitrile):V(water) = 9:1, containing 0.2% acetic acid), using the following gradient elution program for separation: 0–16 min, 12–14% (B); 16–18 min, 14% (B); 18–30 min, 14–16% (B); 30–36 min, 16–20% (B); 36–46 min, 20–24% (B); 46–56 min, 24–30% (B); 56–66 min, 30–50% (B); 66–70 min, 50% (B); 70–80 min, 50–12% (B); and 80–85 min, 12% (B).

The column temperature of organic acid was 45 °C and the detection wavelength was 210 nm. The mobile phase consisted of solvent A (0.02 mol/L K_2_HPO_4_, adjusted pH to 2.3 by H_3_PO_4_ solution), and solvent B (methanol), using the following isocratic elution program for separation: V(A):V(B) = 99:1.

### 2.3. Experimental Grouping

A total of 320 male ICR mice (9 weeks old) were randomly divided into eight large groups (32 groups, 10 mice in each group). The gavage was calculated according to the daily drinking amount equivalent to a body weight of 60 kg to simulate humans (Table S1). After 90 days of continuous intragastric administration, the mice were fasted for 12 h before sacrifice and were free to drink water. Blood was collected from the orbit on the second day, and the mice were killed by cervical dislocation. The sacrificed mice were dissected, and the liver tissues were immediately taken out; a part of the liver was fixed in 4% polyformic acid solution for histopathological research, and the rest was stored at −80 °C for subsequent experiments.

### 2.4. Determination of Serum and Liver Indicators

The mice mentioned in 2.3 were taken for the determination of subsequent indicators:

(1) The mice were weighed before blood collection, and the whole liver and spleen were taken after dissection for weighing. The liver index and spleen index were calculated by the following equations: liver index = liver weight/body weight × 100% and spleen index = spleen weight/body weight × 100%.

(2) Serum preparation: The collected blood samples were centrifuged at 1500× *g* for 15 min at 4 °C, and the serum was separated and stored at –80 °C.

(3) Liver homogenate preparation: A total of 0.5 g of liver was fully ground with a homogenizer and placed in saline to prepare 10% (*m*/*v*) liver homogenate and was then centrifuged at 3000× *g*/min at room temperature for 15 min, after which the supernatant was taken.

(4) The determination of serum AST, ALT, TC, TG, ALP, TBIL, SOD, and T-AOC, and the determination of liver homogenate CAT, GSH, SOD, and MDA were measured strictly in accordance with the kits’ instructions.

(5) Tissue section preparation: Fixed liver tissues were dehydrated using a series of ethanol solutions, embedded in paraffin, and sectioned at 5–6 μm.

(6) Hematoxylin–eosin (HE) staining: HE staining was performed, including dewaxing, immersion, staining, differentiation, blue promotion, dehydration, transparency, etc. After staining, we observed with a microscope.

(7) Oil red (OR) staining: OR staining was performed to evaluate fat deposition. The method was consistent with Do et al. (2021) [20]. Image Pro Plus 6.0 software was used to analyze each photo to obtain the ratio of the red fat droplet area to the total tissue area, i.e., the percentage of the oil red area.

### 2.5. Analysis of Residual Copper in Mouse Liver

Inductively coupled plasma mass spectrometry (ICP-MS) (Agilent 7500A, USA) was used to determine the copper residue in mouse liver tissue [21]. A total of 0.5 g of liver was placed in a 50 mL triangulated flask and baked in the oven (105 °C) until it reached a constant weight. A total of 5 mL 65% HNO_3_ (premium pure) and 2 mL 70% HClO_4_ (premium pure) was added after transferring to the fume hood, heated at 80 °C and 120 °C for 2 h, respectively, and then heated to 190 °C until there was no white smoke in the triangle flask, and the solution became colorless or slightly light yellow. After cooling to room temperature, they were transferred and filtered with ultrapure water to a 25 mL volumetric flask with a constant volume. After passing through a 0.45 µm water-based filter membrane, they proceeded directly to ICP-OES determination by using full quantitative analysis. Results are automatically calculated from the standard curve.

### 2.6. Statistical Analysis

A one-way analysis of variance (ANOVA) and Duncan multiple range tests were conducted to determine the significance of the differences between groups using DPS software (version 7.05), and the experimental data were expressed as the mean values ± standard deviation (SD). The significance analysis within the group was performed by a *t*-test.

## 3. Results

### 3.1. Chemical Composition of Intragastric Fluids

The main components of intragastric fluids, as shown in Table 1, and as previously reported [22], showed the wine was rich in phenolics and organic acids. The dominant phenolics were epicatechin, rutin, ethyl 4-hydroxybenzoate, catechin, isoquercitrin, gentisic acid, quercitrin and epigallocatechin, which accounted for 78.06% of total phenolics. The main organic acid compounds were tartaric acid, lactic acid, and succinic acid, which accounted for 93.46%. With the addition of copper sulfate, the composition of wine changed significantly, but there was no uniform rule. This may be because the molecular interaction between copper ion and each nutrient was very different. However, in general, the results indicated that wine contains many bioactive substances that may promote health.

### 3.2. Serum Indicators of Mice

The duration of the intragastric administration was 90 days. There were 320 mice in the initial gavage and 301 mice at the end of the gavage.

#### 3.2.1. AST and ALT

**Figure 1:**
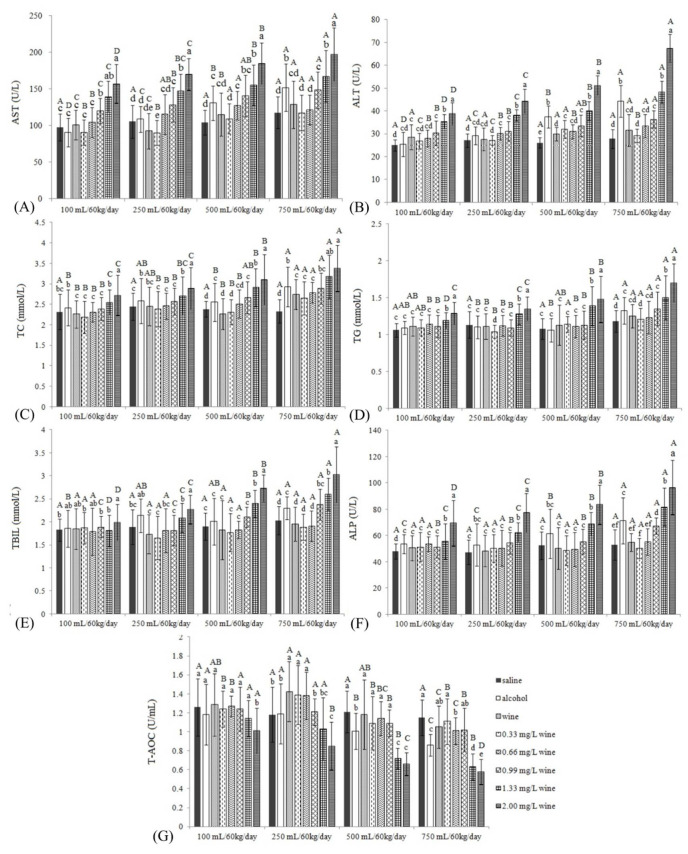
The AST (**A**), ALT (**B**), TC (**C**), TG, (**D**) TBIL (**E**), ALP (**F**), and T-AOC (**G**) levels in the serum of mice. Different capital letters indicate significant differences between treatment groups, and different lowercase letters indicate significant differences within the same dose groups (*p* < 0.05).

The results of AST activity and ALT activity are shown in Figure 1A,B. Firstly, when the daily intragastric doses were low (100 and 250 mL/60 kg/day), there were no significant differences in AST values between alcohol groups (AGs) and saline groups (SGs) (*p* > 0.05). When the dose continued to increase to 500 mL/60 kg/day or 750 mL/60 kg/day, the AST of AG was significantly higher than that of SG, indicating that the animal model was successfully established. Secondly, at intragastric doses of 500 and 750 mL/60 kg/day, the AST values of the wine groups (WGs) were significantly lower than those of AGs, and there were no significant differences from the SGs. Thirdly, when treated with low concentrations of copper (0.33–0.66 mg/L), regardless of the dose, the serum AST values were all significantly lower than those of WGs (*p* < 0.05), and even lower than those of SGs (0.66 mg/L copper, 250 mL/60 kg/day). Fourthly, when the copper concentration rose to 0.99 mg/L (the highest limit of China’s national standard, GB/T 15038-2006), the AST values began to rise, which were significantly higher than those of SGs, and even significantly higher than those of AGs (100 and 250 mL/60 kg/day), which indicated that liver damage in mice had been caused. In addition, when the copper content in wine exceeded the standard (1.33 and 2.00 mg/L), the serum AST values were significantly increased under all doses (*p* < 0.05). In fact, when the intragastric dose of 2.00 mg/L copper concentration was 750 mL/60 kg/day, the AST value had increased to nearly twice as high as that of the SG (Figure 1A).

Changes in ALT levels were similar to AST (Figure 1B). First of all, there were no significant differences in ALT values between AGs and SGs at low doses, but ALT values increased significantly at high doses, indicating that the animal model was successfully established. Secondly, under high-dose conditions, the ALT values of WGs were significantly lower than those of AGs (*p* < 0.05) and either significantly different from those of SG (750 mL/60 kg/day) or close to those of SG (500 mL/60 kg/day), indicating that wine had a certain protective effect. Thirdly, in contrast to AST, the ALT values of low concentrations of copper were slightly lower than those of WGs, but there were basically no significant changes (*p* > 0.05). Fourthly, the serum ALT values of mice were significantly increased at all intragastric doses when the copper content exceeded the standard (1.33 and 2.00 mg/L).

#### 3.2.2. TC and TG

After ethyl alcohol entered the body, the free radicals produced by it attacked hepatocytes, resulting in the changes in TC and TG in the liver (Figure 1C,D). Firstly, at a dose of 250 mL/60 kg/day, AG had already begun to cause a significant increase in TC level (*p* < 0.05), and the increase was positively correlated with the intragastric doses, which was similar to the findings of Li et al. (2015) (Figure 1C) [5]. Secondly, the TC values of WGs were significantly lower than the AG values (except for 250 mL/60 kg/day), and there were no significant differences between WGs and SGs at 100–500 mL/60 kg/day (*p* > 0.05), which showed that functional substances in wine could inhibit the accumulation of TC in the liver to a certain extent. Thirdly, the low concentration of copper (0.33 and 0.66 mg/L) had little effect on TC under all intragastric conditions, which were not much different from the WG. Fourthly, at 100–500 mL/60 kg/day, the TC values of wine with 0.99 mg/L copper were not different from SGs. When it increased to 750 mL/60 kg/day, the TC values were significantly higher than those of SGs. Finally, excessive copper (1.33 and 2.00 mg/L) could significantly increase the serum TC values under almost all doses (except 1.33 mg/L copper, 100 mL/60 kg/day) (*p* < 0.05).

As for serum TG (Figure 1D), firstly, in contrast to TC, the TG level of AG increased significantly when the dose reached 750 mL/60 kg/day (*p* < 0.05). Secondly, under almost all intragastric conditions (except 500 mL/60 kg/day), there were no significant differences in TG levels among WGs, AGs and SGs (*p* > 0.05). Others were basically consistent with the effect on TC (Figure 1C,D).

#### 3.2.3. TBIL and ALP

For mouse TBIL (Figure 1E), first, AG was not significantly higher than SG until the dose reached 750 mL/60 kg/day (*p* < 0.05). Secondly, the TBIL values of WGs were not significantly different from those of SGs under all intragastric conditions (*p* > 0.05), and they were significantly lower than those of AGs except for in the 100 mL/60 kg/day group. Thirdly, low concentrations of copper (0.33 and 0.66 mg/L) were not significantly different from the WGs under all intragastric doses (*p* > 0.05). Fourthly, there were no significant differences between 0.99 mg/L copper wine groups and WGs, and when the intragastric dose was increased to 500 or 750 mL/60 kg/day, the TBIL value was significantly different from that of SG and WG, but there was no significant difference with AG (*p* > 0.05). Finally, when the copper concentration was 1.33 mg/L, TBIL values increased significantly when the doses increased to 500 and 750 mL/60 kg/day, which were significantly different from AGs (*p* < 0.05). When the copper concentration was 2.00 mg/L, TBIL values were significantly increased under all doses, and were significantly higher than those of the AGs at 500 and 750 mL/60 kg/day. 

For ALP (Figure 1F), first of all, all AGs increased ALP, and there was a significant difference between AG and SG at 750 mL/60 kg/day (*p <* 0.05). Secondly, the ALP values of WGs and the low-concentration copper group were not significantly different from those of SGs under all doses (*p* > 0.05) and were significantly lower than those of AG at 750 mL/60 kg/day. Thirdly, only 0.99 mg/L copper at a dose of 750 mL/60 kg/day caused the serum ALP levels of mice to be significantly higher than that of SG. Finally, under all intragastric conditions, excessive concentrations of copper would significantly increase the ALP values of mice (*p <* 0.05). When the concentration was 2.00 mg/L, the ALP value even reached nearly twice that of SG at 750 mL/60 kg/day.

#### 3.2.4. T-AOC

It could be seen from Figure 1G that, firstly, when the intragastric dose was 500 mL/60 kg/day, AG had begun to significantly reduce the serum T-AOC level of mice (*p* < 0.05). Secondly, WG significantly increased the T-AOC level at a dose of 250 mL/60 kg/day, indicating that an appropriate amount of wine was beneficial to health. Under other doses, T-AOC levels in WGs were also significantly higher than those in AGs. Thirdly, compared with WGs, there were no significant differences in T-AOC levels when the copper concentration was ≤0.99 mg/L (*p* > 0.05). In the end, excessive copper concentrations (1.33 and 2.00 mg/L) significantly reduced T-AOC levels under all doses, which was consistent with the report by Araya et al. (2012) that long-term high copper loading would lead to a reduction in relevant antioxidant capacity indicators [23].

### 3.3. Mouse Liver Indicators

#### 3.3.1. Liver Index and Spleen Index

**Figure 2:**
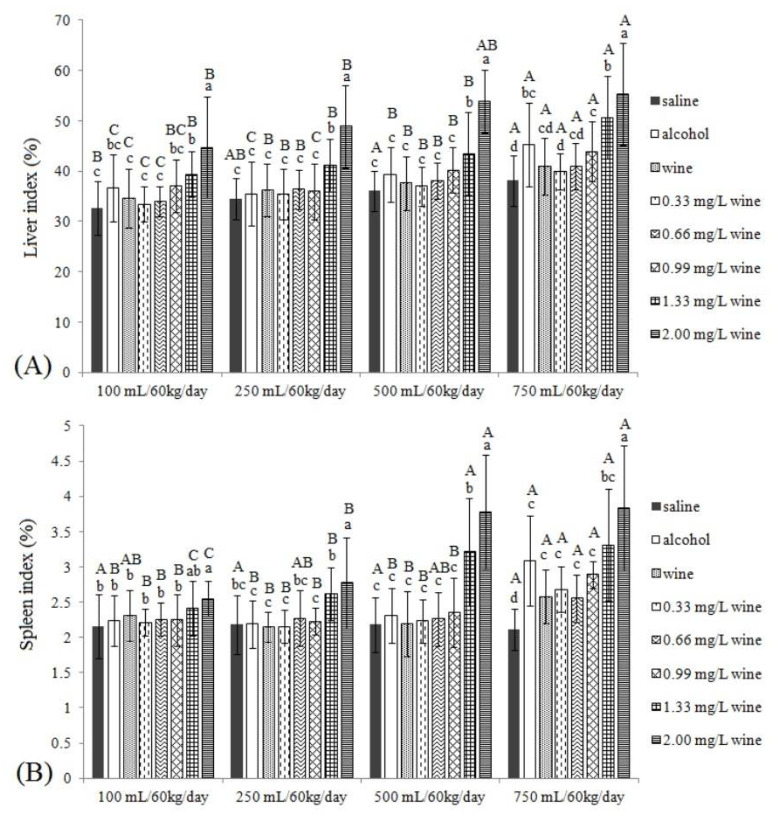
The liver indices (**A**) and spleen indices (**B**) of mice. Different capital letters indicate significant differences between treatment groups, and different lowercase letters indicate significant differences within the same dose groups (*p* < 0.05).

When the liver and spleen are injured, they will swell significantly, resulting in an increase in the liver and spleen index [24]. For the liver index (Figure 2A), it can be found that, firstly, when the intragastric dose was ≥500 mL/60 kg/day, the liver indices of AGs were significantly higher than those of SGs (*p* < 0.05). Secondly, there were no significant differences in liver indices among all AGs and SGs (*p* > 0.05). Thirdly, there were no significant differences in liver indices between low-concentration copper groups (0.33 and 0.66 mg/L) and wine groups (*p* > 0.05). Fourthly, the liver indices were also significantly higher than those of SGs when the doses of copper (0.99 mg/L) were ≥500 mL/60 kg/day. Finally, when the copper concentration was excessive, the liver indices increased significantly at all intragastric doses.

The spleen index was similar to the liver index (Figure 2B) with slight differences. When the dose reached 750 mL/60 kg/day, the spleen index of the AG was significantly higher than that of SG (*p* < 0.05). At low and medium doses (≤500 mL/60 kg/day), the spleen indices of WGs, copper concentration groups and 0.99 mg/L copper concentration groups were not significantly different from SGs (*p* > 0.05). When it rose to 750 mL/60 kg/day, the three kinds of groups were significantly elevated and significantly different from SGs, indicating that all the spleens were enlarged. Moreover, excessive copper almost caused splenomegaly in all treated mice (except at 100 and 250 mL/60 kg/day at 1.33 mg/L).

#### 3.3.2. CAT, GSH, SOD and MDA

**Figure 3:**
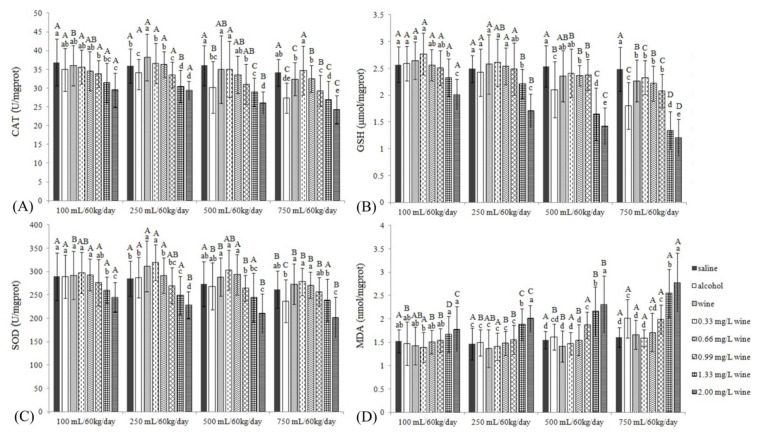
The CAT (**A**), GSH (**B**), SOD (**C**) and MDA (**D**) levels in mouse livers. Different capital letters indicate significant differences between treatment groups, and different lowercase letters indicate significant differences within the same dose groups (*p* < 0.05).

By analyzing the CAT levels of mice in different groups (Figure 3A), it was observed that, firstly, when the dose reached 500 mL/60 kg/day, the CAT value began to decrease significantly (*p <* 0.05). Secondly, the CAT values of WGs were not significantly different from those of SGs or were higher than those of SGs (250 mL/60 kg/day), and they were significantly higher than those of AGs in the 500 and 750 mL/60 kg/day groups. Thirdly, the low-concentration copper groups (0.33 and 0.66 mg/L) were not significantly different from SGs under all dosage conditions (*p* > 0.05), but the CAT level was significantly higher than that of WG at 750 mL/60 kg/day. Fourthly, CAT values of 0.99 mg/L copper groups were significantly lower than those of SGs under all intragastric dose conditions, indicating that the liver antioxidant system was damaged. Fifthly, excessive copper impaired the antioxidant system and reduced CAT levels.

The content of GSH could reflect the activity of glutathione peroxidase (GPx) (Figure 3B). Changes in GSH levels were similar to changes in CAT levels, except for that, firstly, the GSH value of WG was lower than that of SG at 750 mL/60 kg/day; secondly, the three groups with a copper concentration of 0.99 mg/L and below were significantly lower than SGs when the intragastric dose was increased to 750 mL/60 kg/day, but significantly higher than AG (*p* < 0.05). In addition, the GSH values of the 1.33 and 2.00 mg/L copper groups were only 54% and 48% of that for SG, respectively, which reflected that the liver damage was very serious.

The SOD levels are shown in Figure 3C. Firstly, AGs only started to cause a significant decrease in liver SOD levels at 750 mL/60 kg/day. Secondly, there were almost no significant differences among WGs, the medium or low copper concentration groups (0.33, 0.66, 0.99 mg/L) and SGs (*p* > 0.05), and their SOD levels were significantly higher than those of AG at 750 mL/60 kg/day. Thirdly, all the excess copper concentration (1.33 and 2.00 mg/L) groups significantly reduced in SOD levels (*p* < 0.05).

MDA levels, as the final product of lipid peroxidation, are shown in Figure 3D. Firstly, as with SOD, the MDA levels in AGs began to increase significantly at 750 mL/60 kg/day (*p <* 0.05). Secondly, the MDA contents of WGs and low-concentration copper (0.33 and 0.66 mg/L) groups were not significantly different from SGs (*p* > 0.05) and were lower than those of AGs at 750 mL/60 kg/day. Thirdly, the 0.99 mg/L copper concentration groups under the medium- and high-dose conditions (500, 750 mL/60 kg/day) showed a rise in MDA levels, becoming significantly higher than those of SGs. Fourthly, 1.33 and 2.00 mg/L copper concentration groups significantly increased the MDA levels of mice when the intragastric dose was ≥250 mL/60 kg/day. This indicated that the effects of ethanol and copper on the accumulation of MDA in the liver have exceeded the antagonistic effect of wine polyphenols, which is similar to the results of Ozcelik et al. (2003) [25].

### 3.4. Histopathological Observation of Mouse Liver 

#### 3.4.1. HE Staining Results

**Figure 4:**
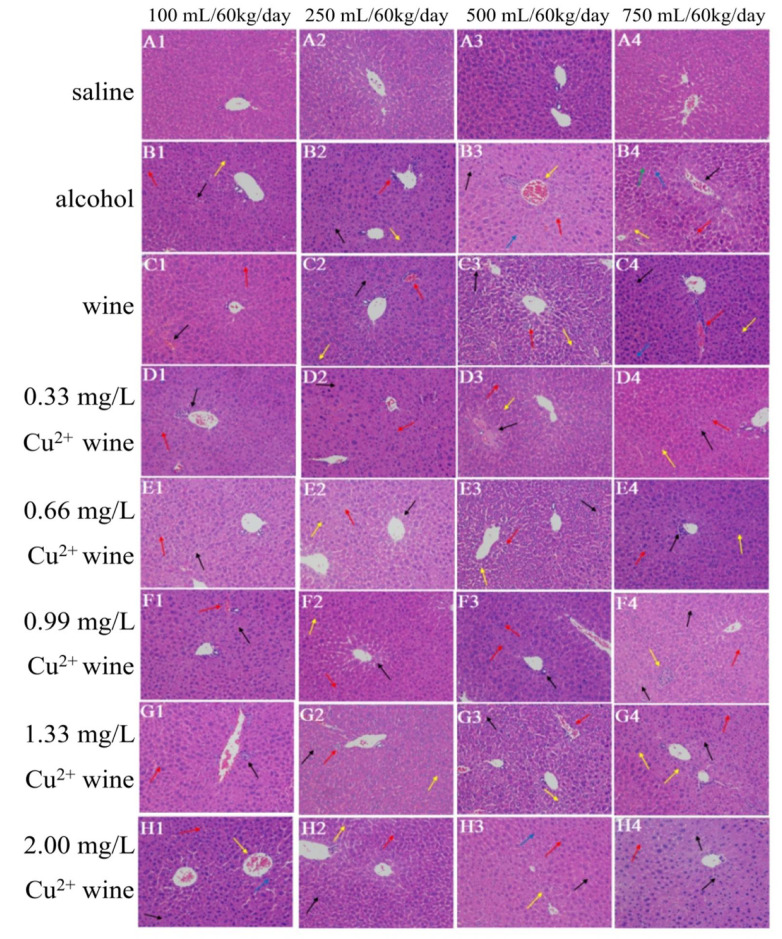
The HE staining of mouse livers with 200 times magnification.

HE staining can make chromatin and ribosomes appear purple-blue, and components in cytoplasm and extracellular matrices appear red. It is one of the most commonly used staining methods in morphology [26]. By observing the HE staining results of liver tissue sections (Figure 4(A1–A4)), it could be seen that the liver tissue structure in the SGs were complete, with clear hepatic lobules, clear cell boundaries, round and clear nuclei, and abundant cytoplasm. The hepatic cords were neatly arranged, the hepatic sinusoids were normal, the tissue had no obvious inflammation, necrosis and fibrosis, and the overall structure was normal.

In the AGs, the low-dose group (100 mL/60 kg/day) had fewer tissue lesions (Figure 4(B1)), a clear structure of the hepatocyte cord, and vacuoles of different sizes could be seen in some hepatocytes, as shown by the black arrow. Lymphocytes could be seen in the tissue, as shown by the red arrow, and binuclear hepatocytes could be seen in the tissue, as shown by the yellow arrow. However, when the intragastric dose increased, the tissue pathology became worse. At 750 mL/60 kg/day (Figure 4(B4)), the hepatocyte cord structure was disordered, and red blood cells could be seen in the venous cavity, as shown by the black arrow. Hepatocytes had mild necrosis, as shown by the red arrows. Mild dilatation and congestion could be seen in the hepatic sinuses, as shown by the yellow arrow. There was lymphocyte infiltration in the tissue, as shown by the green arrow. Binuclear hepatocytes could be seen in the tissue, as shown by the blue arrow.

Compared with AGs, the WGs (Figure 4(C1–C4)) had relatively lighter lesions. At 100 mL/60 kg/day (Figure 4(C1)), the hepatocyte cord structure was still clear, and the cells did not show obvious degeneration, and there was dilated congestion in the hepatic sinuses, as shown by the black arrow. Binuclear hepatocytes could be seen in the tissue, as shown by the red arrow. At 750 mL/60 kg/day (Figure 4(C4)), the arrangement of the hepatocyte cord was slightly disordered, and Kupffer cells could be seen in the liver sinusoids, as shown by the black arrow. A few erythrocytes were seen in the portal area, as indicated by the red arrow. There were fatty vacuoles of different sizes in hepatocytes (as shown by the yellow arrow), and binuclear hepatocytes could be found in the tissue (as shown by the blue arrow).

Similar to the aforementioned serum indicators and liver indicators, the low-concentration copper (0.33 and 0.66 mg/L) groups (Figure 4(D1–E4)) had consistent pathological changes to WGs. The pathological changes were mild at low doses. With the increase in the dose, the disease gradually worsened, but it was significantly better than that in AGs. At the highest limit of China’s national standard for copper concentration (0.99 mg/L) (Figure 4(D1–E4)) at a low intragastric dose, the pathological changes were similar to that of low-concentration copper, but when the dose was higher, the pathological changes deepened. At 750 mL/60 kg/day (Figure 4(C4)), the hepatocytes were swollen, the cytoplasm was loose, and vacuoles of different sizes could be found in some liver cells, as shown by the black arrows. Hepatocyte nuclei dissolved and were vacuolated, as shown by the red arrow, and small foci of inflammatory necrosis could be seen in the tissue, as shown by the yellow arrow.

The lesions in the excessive copper (1.33 and 2.00 mg/L) groups (Figure 4(G1–H4)) were significantly higher than those in the other groups, which had certain similarities to the rat liver injury induced by cadmium sulfide nanoparticles [27], especially at 750 mL/60 kg/day. The hepatic sinus space in the 1.33 mg/L copper group (Figure 4(G4)) became narrower, a large number of hepatocyte cytoplasm became loose, and vacuoles of different sizes were seen in the cells, showing watery degeneration and steatosis, as shown by the black arrow. There was a small amount of lymphatic infiltration in the tissue, as shown by the red arrow, and multiple binuclear hepatocytes could be seen in the tissue, as shown by the yellow arrows. When the copper concentration was 2.00 mg/L (Figure 4(H4)), the hepatic sinusoids became narrow and disappeared, a large number of hepatocytes were swollen, the cytoplasm was loose, and vacuoles of various sizes were seen in some hepatocytes, as shown by the black arrow. There was a small amount of lymphocyte infiltration in the tissue, as marked by the red arrow.

#### 3.4.2. OR Staining Results

**Figure 5:**
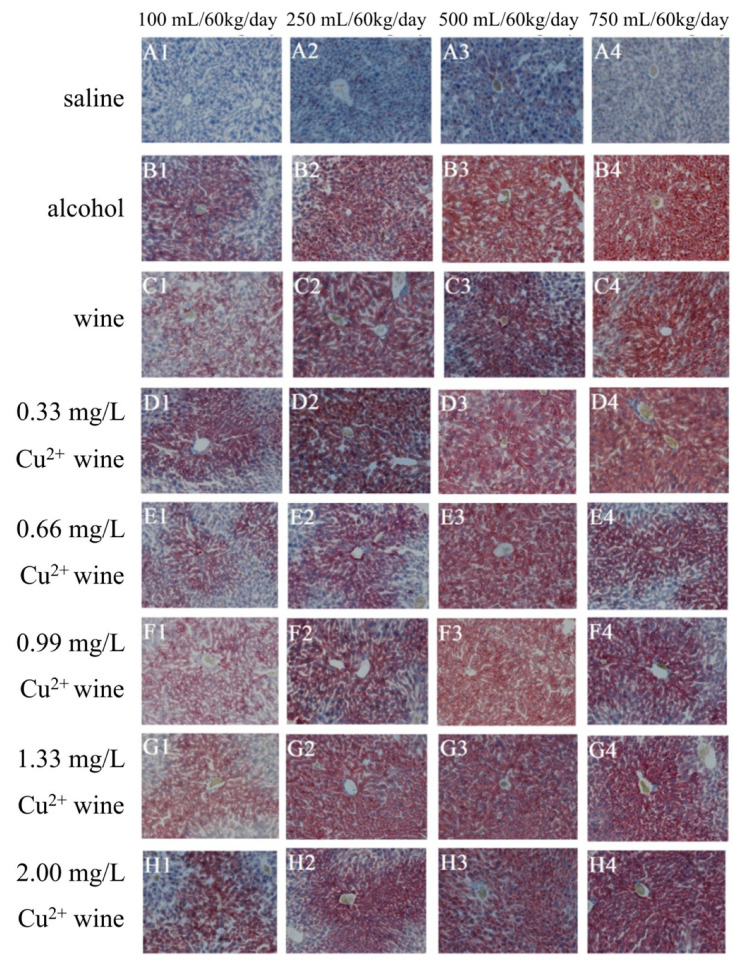
The oil red staining of mouse livers with 200 times magnification.

Liver sections were further prepared for OR staining observation (Figure 5 and Table S2). The darker the color, the greater the percentage of OR, indicating the more lipids in the tissue. In the AGs, the significant increase in the staining area of the liver could be observed at 100 mL/60 kg/day, and it increased significantly with the dose; however, the areas of WGs were significantly lower than those of AGs, and the WGs and low-concentration copper (0.33 and 0.66 mg/L) groups were not much different from SGs. The staining areas of the 0.99 mg/L copper group under medium- and high-dose conditions (500 and 750 mL/60 kg/day) were significantly higher than those of WGs, and the over-standard copper concentration groups (1.33 and 2.00 mg/L) increased the staining areas under almost all intragastric administrations (except for 1.33 mg/L, 100 mL/60 kg/day).

### 3.5. Analysis of Copper Residue in Mouse Liver

The copper residue in mouse liver was analyzed, and the results are shown in Table 2. The liver copper contents of mice in SGs were about 2 μg/g, and there were no significant differences between AGs and SGs. All concentrations of copper significantly increased the liver copper in mice (*p* < 0.05), and the liver copper concentration was positively correlated with the daily intragastric dose and the copper concentration in wine. When the copper exceeded GB 2-fold, the liver copper concentration reached 3.34 ± 0.12 μg/g under the condition of the 750 mL/60 kg/day dose for 3 months, which was 1.69 times that of SG.

## 4. Discussion

Serum AST, ALT, TC, TG, TBIL and ALP activity levels were key medical indicators for evaluating and testing liver function, and variations in these levels were closely linked to the occurrence of liver diseases [3,24]. For a long time, serum ALT and AST enzymatic activities had been regarded as the most sensitive indicators of hepatic injury, because the damage of hepatocytes would change their transport functions and membrane permeability, resulting in the leakage of enzymes from the cells [24]. Based on the data of various serum indicators, the following could be concluded: (1) Ethyl alcohol intake was significantly correlated with serum indicators in mice, and 500 mL/60 kg/day caused the deterioration of most serum indicators. (2) Wine could significantly reduce the hepatic injury caused by ethyl alcohol in mice. (3) Low concentrations of copper (0.33 and 0.66 mg/L) had little effect on serum indicators related to hepatic injury, but significantly improved the serum AST of mice. (4) Under the condition of long-term intake, wine with a copper concentration of 0.99 mg/L (in line with China’s national standard) would still cause damage to the liver of mice, especially when the dose was ≥500 mL/60 kg/day. (5) The chronic intake of wine containing excessive copper (1.33 and 2.00 mg/L) led the deterioration of almost all serum indicators, and significantly increased the degree of hepatic injury in mice.

GSH, CAT and SOD are the major components of the antioxidant system of mammalian cells, which support each other to constitute an antioxidant defense system, which could effectively eliminate all kinds of harmful substances generated in the metabolism of the body. SOD is the first line of defense in the antioxidant defense system and could promote the disproportionation of superoxide anion radicals in cells, with H_2_O_2_ as the product. On the other hand, CAT could catalyze H_2_O_2_ to decompose H_2_O and O_2_, preventing the production of more toxic hydroxyl free radicals. Meanwhile, GSH could scavenge free radicals and protect sulfhydryl groups in molecules, such as proteins and enzymes [24,28,29,30]. AG started to significantly reduce the SOD level in the liver of mice when treated with 750 mL/60 kg/day (*p* < 0.05), whereas CAT and GSH had begun to decrease significantly when treated with 500 mL/60 kg/day (Figure 3). It proved that the body first relies on CAT and GSH to resist free radicals, and then SOD. In addition, changes in the activity of enzymes related to antioxidation could reflect the degree of free radicals in the human body, whereas changes in the content of MDA, the final product of lipid peroxidation, could clearly reflect the degree of lipid peroxidation in animals [3,24]. Based on the data of various liver indicators, the following was found: (1) Ethyl alcohol intake is related to liver indicators. When the gavage reached 500 mL/60 kg/day, ethyl alcohol caused liver enlargement in mice and caused most liver biochemical indices to deteriorate, indicating that long-term drinking would cause hepatic injury in mice when the intake of ethyl alcohol was ≥500 mL/60 kg/day. (2) Wine could significantly reduce hepatic injury in mice caused by ethyl alcohol (*p* < 0.05). (3) Low concentrations of copper (0.33 and 0.66 mg/L) had little effect on liver indicators related to hepatic injury. (4) The chronic intake of wine with a copper concentration of 0.99 mg/L (in line with GB) increased the levels of CAT (all gavage) and MDA (medium and high gavage) in mouse liver. (5) Even at a low intragastric dose, the long-term intake of wine containing excessive concentrations of copper (1.33 and 2.00 mg/L) resulted in the deterioration of almost all liver indicators, and significantly aggravated the degree of hepatic injury in mice.

Many studies have shown that excessive alcohol consumption could cause damage and lesions of many organs, including the liver, which is a major contributor to the global burden of disease [1]. It is estimated that improper intake of ethyl alcohol is the third leading cause of early death and disease in the European Union (EU 2006) and causes more than 5% of diseases worldwide (WHO 2020). In this study, the ethyl alcohol intake of all alcoholic beverages was significantly correlated with the degree of hepatic injury in mice, showing a dose-dependent manner, which was consistent with other studies [6,11,31,32].

It has been pointed out that wine might also have potential health benefits when consumed in moderation [31,33]. At the same dose, compared to AG, wine had a certain protective effect on the liver. Tverdal et al. (2018) noted an association between the total amount of drinking and the risk of ALD, but the type of alcoholic beverage was critical for the strength of the association, with wine consumption having a lower risk than beer and liquor [11]. This might be partly due to the various liver-protecting ingredients in wine, such as resveratrol and anthocyanin. The significant reduction in oxidants after drinking wine showed that the moderate and long-term drinking of red wine could prevent lipid peroxidation in the circulation [34,35]. In the human body, copper is mainly absorbed by the gastrointestinal tract and partially excreted with feces, whereas the rest is transported to the liver [36]. Studies have shown that the long-term excessive intake of copper will lead to copper accumulation in the body, especially in the liver [37]. Overmuch copper in the body could cause hemolysis, jaundice and even death [38], and might also cause cancer, diabetes, atherosclerosis, neurological disorders and other diseases [39]. Under the experimental conditions, the liver copper content of mice in the saline group was about 2 μg/g, which was significantly lower than the liver copper concentration reported by Yu et al. (2021), which might be due to the variety difference of the mice [37]. The low concentration of copper (0.33–0.66 mg/L) basically did not aggravate hepatic injury in mice, which was consistent with evidence from the literature research [40], and even significantly improved the serum AST value of mice, which showed that copper could play a positive role in the body within this concentration range. However, it did not increase the T-AOC as reported by He et al. (2014) [41]. This might be due to the fact that multiple variables of the functional components of ethyl alcohol, copper, and wine were considered simultaneously in this study. The beneficial effects of low concentrations of copper were overshadowed by the protective effects of polyphenols in wine. Long-term drinking, especially long-term and heavy drinking (≥500 mL/60 kg/day) of wine with the maximum copper concentration (0.99 mg/L) allowed by China’s national standard was harmful. Moreover, exposure to high concentrations of copper (1.33–2.00 mg/day/L) could promote oxidative stress hepatic injury by increasing the level of liver indicators (AST, ALT, TC, TG, TBIL, ALP) and MDA content, while reducing the activity of antioxidant enzymes (GSH, SOD and CAT). Furthermore, in the pathological examination, the liver inflammation and degeneration symptoms of mice exposed to high-level copper wine were obvious, which is similar to cadmium sulfide-treated rats [27]. At this time, the synergistic damage effect of copper and ethyl alcohol was greater than the protective effect of the functional components in wine, especially polyphenols. This was consistent with previous reports [25,37]. In general, the higher the intake of alcoholic beverages and the higher the copper concentration in wine, the higher the liver copper concentration of mice, indicating that wine with excessive copper would accumulate in the body and destroy the tissues. Yu et al. (2021) suggested that proper dietary copper could improve antioxidant stress levels and improve liver function by promoting mitophagy and copper enzymes that play antioxidative roles, whereas the accumulation of excess copper could induce liver lesions by enhancing apoptosis and inhibiting mitophagy pathways [37]. Saporito-Magriñá et al. (2017a, 2017b) reported that copper could induce mitochondrial dysfunction in mouse liver mitochondria and cause phospholipid peroxidation, which further leads to cell homeostasis disorder and cell death [42,43]. These might explain the conclusion of this study to some extent, but the specific mechanism of the interaction between copper and polyphenols in wine on alcoholic hepatic injury in mice needs to be further studied.

In recent years, copper in wine has exceeded the standard from time to time and has been repeatedly banned. Taking China as an example, from 2014 to 2017, due to excessive copper, customs seized many batches of imported wine from Argentina, Spain, Chile, Cyprus, Ukraine, France, and other countries. Excessive copper content in wine has become one of the main reasons for Chinese customs to seize wine. In the future, it is necessary to further strengthen the control of copper content in wine and the inspection of market wine, so as to protect the health of consumers.

## 5. Conclusions

Overall, copper in wine and polyphenols interacted with ALD in mice. Firstly, when the drinking amount reached 500 mL/60 kg/day (14% abv), mouse livers had a certain degree of hepatic injury (AST 130.71 U/L, ALT 37.45 U/L, TC 2.55 U/L, T-AOC 1.01 U/mL, CAT 30.20 U/mgprot, and GSH 2.10 U/mgprot). Secondly, compared with the AG, wine can well reduce the damage, but when the drinking amount reached 750 mL/60 kg/day, the serum indicators and liver indicators of mice were slightly worsened, indicating that wine had protection against ALD in mice. Thirdly, low concentrations of copper had little effect on hepatic injury, and only significantly improved the mouse serum AST value (0.33 mg/L, 109.21 U/L; 0.66 mg/L, 127.29 U/L). Fourth, when the concentration of copper was 0.99 mg/L, 500 mL/60 kg/day, it began to cause hepatic injury in mice, showing that the hepatic injury of alcohol and/or copper had begun to exceed the protection of the wine’s functional substances (mainly polyphenols). Fifth, high concentrations of copper significantly aggravated ALD in mice. At this time the toxicity of copper had become dominant. 

In recent years, the phenomenon of copper exceeding the standard in wine has appeared from time to time, and it has been repeatedly banned. In the future, it is necessary to further strengthen the control of copper content in wine and the inspection of market wine, so as to protect the health of consumers.

## Figures and Tables

**Table 1:** The phenolic compounds and organic acids of intragastric fluids.

	Wine	0.33 mg/L Copper Wine	0.66 mg/L Copper Wine	0.99 mg/L Copper Wine	1.33 mg/L Copper Wine	2.00 mg/L Copper Wine
Phenolic compounds						
*Flavanol*						
Epigallocatechin	50.28 ± 3.13 d	45.24 ± 0.14 e	52.21 ± 2.23 c	57.67 ± 1.52 a	55.13 ± 6.29 b	58.26 ± 5.71 a
Catechin	143.78 ± 2.56 b	144.24 ± 1.75 b	138.66 ± 4.28 c	153.78 ± 6.56 a	128.74 ± 1.60 d	125.32 ± 2.36 e
Epigallocatechin 3-gallate	12.23 ± 0.55 a	10.23 ± 0.26 b	9.89 ± 1.11 b	9.53 ± 0.99 b	9.44 ± 0.89 b	9.33 ± 1.00 b
Epicatechin	236.72 ± 8.79 a	230.66 ± 6.49 b	228.71 ± 9.23 c	237.21 ± 4.57 a	235.82 ± 8.11 a	226.77 ± 7.84 d
Epicatechin-3-O-gallate	34.21 ± 1.55 a	32.89 ± 2.06 a	30.01 ± 1.66 b	28.99 ± 0.99 b	25.32 ± 2.23 c	25.44 ± 0.60 c
Myricetin	32.46 ± 0.53 a	28.46 ± 1.24 b	27.96 ± 0.39 b	28.04 ± 0.55 b	27.89 ± 0.50 b	28.82 ± 1.12 b
Isorhamnetin	2.33 ± 0.10 a	1.16 ± 0.08 a	1.03 ± 0.12 a	0.99 ± 0.09 a	1.00 ± 0.08 a	1.13 ± 0.15 a
*Flavonol*						
Rutin	168.49 ± 1.85 a	123.43 ± 0.76 f	128.11 ± 4.21 e	146.79 ± 8.37 d	152.41 ± 3.48 c	160.33 ± 2.36 b
Isoquercitrin	108.52 ± 1.22 c	106.22 ± 4.68 d	105.82 ± 5.04 d	109.51 ± 6.11 bc	123.83 ± 0.88 a	111.22 ± 3.49 b
Quercitrin	66.57 ± 4.07 a	58.87 ± 2.88 b	59.11 ± 5.02 b	56.13 ± 4.73 c	56.02 ± 7.01 c	55.49 ± 1.99 c
Resveratrol	4.56 ± 0.22 a	4.44 ± 0.43 a	4.38 ± 0.42 a	4.36 ± 0.39 a	4.39 ± 0.56 a	4.33 ± 0.50 a
Quercetin	29.44 ± 1.67 a	29.23 ± 2.23 a	29.11 ± 0.79 a	29.04 ± 1.98 a	28.89 ± 3.21 a	29.34 ± 2.66 a
Luteolin	1.35 ± 0.04 a	0.95 ± 0.15 a	0.88 ± 0.12 a	0.85 ± 0.10 a	0.83 ± 0.18 a	0.79 ± 0.13 a
Apigenin	0.50 ± 0.03 a	0.48 ± 0.03 a	0.47 ± 0.02 a	0.51 ± 0.03 a	0.46 ± 0.01 a	0.52 ± 0.06 a
Kaempferol	1.28 ± 0.08 a	1.20 ± 0.14 a	1.21 ± 0.10 a	1.17 ± 0.13 a	1.19 ± 0.15 a	1.40 ± 0.09 a
*Hydroxycinnamic acid*						
Trans-fertaric acid	2.49 ± 0.19 a	2.40 ± 0.18 a	2.39 ± 0.43 a	2.32 ± 0.22 a	2.35 ± 0.18 a	2.36 ± 0.18 a
Caffeic acid	18.87 ± 1.43 a	18.67 ± 2.24 a	18.57 ± 1.46 a	18.62 ± 1.79 a	18.55 ± 2.03 a	18.49 ± 2.44 a
Coumaric acid	24.11 ± 1.58 b	25.08 ± 0.59 a	25.11 ± 1.60 a	25.11 ± 1.23 a	25.13 ± 1.11 a	25.12 ± 0.99 a
4-hydroxycinnamic acid	4.99 ± 0.49 c	4.95 ± 0.34 c	4.89 ± 0.21 c	4.97 ± 0.52 c	6.31 ± 0.73 a	5.22 ± 0.55 b
Ferulic acid	4.08 ± 0.11 a	4.05 ± 0.52 a	3.99 ± 0.43 a	3.95 ± 0.17 a	3.78 ± 0.15 a	3.68 ± 0.26 a
3-hydroxycinnamic acid	3.22 ± 0.41 c,d	3.52 ± 0.22 b,c	3.89 ± 0.32 a	3.74 ± 0.44 a,b	3.59 ± 0.65 a,b	3.19 ± 0.16 d
Sinapic acid	10.26 ± 0.14 a	5.26 ± 0.85 c	4.96 ± 1.01 c	4.99 ± 0.88 c	8.24 ± 0.98 b	5.20 ± 0.56 c
Isoferulic acid	4.28 ± 0.08 a	1.28 ± 0.05 b	1.34 ± 0.04 b	1.33 ± 0.06 b	1.19 ± 0.12 b	1.19 ± 0.03 b
*Hydroxy benzoic acid*						
Gallic acid	30.06 ± 2.77 a	30.12 ± 1.88 a	29.96 ± 3.10 a	30.10 ± 2.70 a	29.69 ± 2.42 a	31.26 ± 2.55 a
Protocatechuic acid	0.79 ± 0.11 a	0.82 ± 0.08 a	0.85 ± 0.03 a	0.86 ± 0.09 a	0.83 ± 0.15 a	0.84 ± 0.10 a
Gentisic acid	96.56 ± 7.29 a	96.16 ± 6.63 a	95.86 ± 6.37 a	95.12 ± 9.21 a	92.54 ± 3.03 b	90.88 ± 8.30 b
Vanillic acid	37.98 ± 0.98 a,b	39.59 ± 2.99 a	37.30 ± 1.23 b	36.98 ± 3.37 b	37.11 ± 0.55 b	37.03 ± 1.00 b
Syringic acid	15.87 ± 0.23 a	16.87 ± 0.64 a	16.99 ± 0.27 a	17.01 ± 0.98 a	17.68 ± 1.10 a	16.87 ± 0.98 a
Methyl 3,4-dihydroxybenzoate	6.44 ± 0.22 a	5.68 ± 0.42 a	5.44 ± 0.33 a	5.38 ± 0.45 a	5.37 ± 0.24 a	5.23 ± 0.44 a
Methyl vanillate	1.42 ± 0.08 a	1.52 ± 0.14 a	1.33 ± 0.10 a	1.29 ± 0.08 a	1.31 ± 0.12 a	1.25 ± 0.10 a
Ethyl 4-hydroxybenzoate	165.93 ± 9.33 d	168.23 ± 7.27 c	170.63 ± 11.03 b	171.22 ± 8.00 a,b	173.02 ± 6.49 a	172.99 ± 5.98 a
Vanilic acid ethyl ester	8.19 ± 0.63 a	8.49 ± 0.54 a	8.52 ± 0.60 a	8.66 ± 0.92 a	8.55 ± 0.81 a	8.53 ± 0.63 a
Organic acids						
Oxalic acid	11.10 ± 0.03 b,c	11.16 ± 0.04 b,c	10.88 ± 0.12 c	11.28 ± 0.02 a,b	11.47 ± 0.06 a	10.56 ± 0.33 d
Tartaric acid	2181.40 ± 30.10 b	2186.78 ± 15.31 b	2276.53 ± 25.14 a	2256.91 ± 8.93 a	2251.93 ± 3.53 a	2215.29 ± 16.63 b
Quinic acid	30.08 ± 1.01 a	29.03 ± 1.00 a	28.91 ± 2.01 a	29.01 ± 1.00 a	29.08 ± 3.26 a	28.22 ± 0.25 a
Pyruvic acid	80.35 ± 0.01 a	80.00 ± 1.00 a	80.84 ± 2.00 a	78.36 ± 2.10 a	82.02 ± 4.02 a	76.01 ± 6.01 a
Shikimic acid	102.98 ± 3.00 a	103.07 ± 3.00 a	106.12 ± 4.01 a	100.86 ± 10.52 a	101.69 ± 0.60 a	100.49 ± 5.50 a
Malic acid	107.05 ± 7.00 a	112.05 ± 2.01 a	107.86 ± 3.01 a	110.02 ± 4.00 a	110.61 ± 4.60 a	105.18 ± 10.01 a
Malonic acid	6.99 ± 0.50 a,b	6.85 ± 0.15 a,b	6.74 ± 0.24 a,b	6.95 ± 0.05 a,b	7.22 ± 0.26 a	6.42 ± 0.39 b
Lactic acid	2101.15 ± 51.00 a	2080.52 ± 20.02 a	2036.01 ± 14.00 a,b	1974.30 ± 74.01 b	2042.15 ± 42.18 a,b	2019.42 ± 19.53 a,b
Citric acid	25.79 ± 0.88 a	26.01 ± 1.04 a	26.45 ± 2.45 a	26.42 ± 4.42 a	26.10 ± 1.10 a	26.28 ± 1.28 a
Fumaric acid	1.1 ± 0.20 a	0.93 ± 0.07 a	1.11 ± 0.14 a	1.05 ± 0.05 a	0.99 ± 0.01 a	1.07 ± 0.13 a
Succinic acid	943.11 ± 43.00 a	938.09 ± 47.99 a	930.23 ± 30.01 a	928.26 ± 22.07 a	928.22 ± 31.50 a	926.62 ± 19.52 a

Values with different letters are significantly different (*p* < 0.05) from each other.

**Table 2:** The copper content in liver of mouse.

Groups	Copper Content in Mouse Liver (μg/g)			
	Drinking Amount (mL/60 kg/day)			
	100	250	500	750
saline	1.98 ± 0.22 ^a E^	2.03 ± 0.25 ^a E^	2.05 ± 0.21 ^a E^	1.98 ± 0.27 ^a E^
alcohol	2.01 ± 0.34 ^a E^	1.94 ± 0.23 ^a E^	2.13 ± 0.33 ^a E^	1.89 ± 0.32 ^a E^
wine	2.09 ± 0.22 ^a E^	2.02 ± 0.31 ^a E^	2.09 ± 0.24 ^a E^	1.99 ± 0.29 ^a E^
0.33 mg/L copper wine	2.13 ± 0.29 ^b DE^	2.18 ± 0.31 ^ab D^	2.26 ± 0.24 ^a DE^	2.33 ± 0.23 ^a D^
0.66 mg/L copper wine	2.24 ± 0.23 ^b D^	2.34 ± 0.19 ^ab CD^	2.39 ± 0.28 ^a D^	2.45 ± 0.31 ^a D^
0.99 mg/L copper wine	2.48 ± 0.55 ^c C^	2.59 ± 0.33 ^bc C^	2.67 ± 0.14 ^b C^	2.81 ± 0.34 ^a C^
1.33 mg/L copper wine	2.69 ± 0.29 ^d B^	2.82 ± 0.29 ^c B^	2.96 ± 0.23 ^b B^	3.15 ± 0.11 ^a B^
2.00 mg/L copper wine	2.98 ± 0.22 ^d A^	3.14 ± 0.13 ^c A^	3.29 ± 0.19 ^b A^	3.34 ± 0.12 ^a A^

Different capital letters indicate significant differences between treatment groups, and different lowercase letters indicate significant differences within the same dose groups (*p* < 0.05).

## Data Availability

The original contributions presented in the study are included in the article/Supplementary Material, further inquiries can be directed to the corresponding author/s.

## Supplementary Materials

The following supporting information can be downloaded at: https://www.mdpi.com/article/10.3390/foods11162383/s1, Table S1: Experimental animal grouping; Table S2: The oil red staining area in liver of mouse.
