# Supplementary material for: Interactive Effects of Copper and Functional Substances in Wine on Alcoholic Hepatic Injury in Mice
Source: Foods. 2022 Aug 9;11(16):2383. doi: 10.3390/foods11162383 (PMC9407149; doi:10.3390/foods11162383)
Supplement: Supplementary file 1 [file foods-11-02383-s001.zip › foods-1832202-supplementary.pdf]

Supplementary Materials

Table S1. Experimental animal grouping.

Groups	Drinking amount (mL/60kg/day)			
	100	250	500	750
saline	Treatment (1)	Treatment (2)	Treatment (3)	Treatment (4)
alcohol	Treatment (5)	Treatment (6)	Treatment (7)	Treatment (8)
wine	Treatment (9)	Treatment (10)	Treatment (11)	Treatment (12)
0.33 mg/L copper wine	Treatment (13)	Treatment (14)	Treatment (15)	Treatment (16)
0.66 mg/L copper wine	Treatment (17)	Treatment (18)	Treatment (19)	Treatment (20)
0.99 mg/L copper wine	Treatment (21)	Treatment (22)	Treatment (23)	Treatment (24)
1.33 mg/L copper wine	Treatment (25)	Treatment (26)	Treatment (27)	Treatment (28)
2.00 mg/L copper wine	Treatment (29)	Treatment (30)	Treatment (31)	Treatment (32)

Table S2. The oil red staining area in liver of mouse.

Groups	Area (%)			
	100 mL/60kg/day	250 mL/60kg/day	500 mL/60kg/day	750 mL/60kg/day
saline	0.009	0.304	0.152	0.35
alcohol	8.289	36.895	41.242	49.68
wine	7.809	22.74	25.271	34.886
0.33 mg/L copper wine	8.743	24.357	25.888	35.712
0.66 mg/L copper wine	8.111	23.975	28.959	35.045
0.99 mg/L copper wine	8.181	24.569	35.443	39.425
1.33 mg/L copper wine	8.901	28.075	39.784	48.766
2.00 mg/L copper wine	11.855	39.839	42.217	66.558
